# Optimal Skin Cancer Detection Model Using Transfer Learning and Dynamic-Opposite Hunger Games Search

**DOI:** 10.3390/diagnostics13091579

**Published:** 2023-04-28

**Authors:** Abdelghani Dahou, Ahmad O. Aseeri, Alhassan Mabrouk, Rehab Ali Ibrahim, Mohammed Azmi Al-Betar, Mohamed Abd Elaziz

**Affiliations:** 1 Mathematics and Computer Science Department, University of Ahmed DRAIA, Adrar 01000, Algeria; 2 Department of Computer Science, College of Computer Engineering and Sciences, Prince Sattam Bin Abdulaziz University, Al-Kharj 11942, Saudi Arabia; 3 Mathematics and Computer Science Department, Faculty of Science, Beni-Suef University, Beni-Suef 65214, Egypt; 4 Department of Mathematics, Faculty of Science, Zagazig University, Zagazig 44519, Egypt; rehab100r@yahoo.com; 5 Artificial Intelligence Research Center (AIRC), College of Engineering and Information Technology, Ajman University, Ajman P.O. Box 346, United Arab Emirates; 6 Faculty of Computer Science & Engineering, Galala University, Suez 43511, Egypt; 7 Department of Electrical and Computer Engineering, Lebanese American University, Byblos 10999, Lebanon

**Keywords:** medical diagnosis, skin cancer, Hunger Games Search (HGS), Particle Swarm Optimization (PSO), deep learning

## Abstract

Recently, pre-trained deep learning (DL) models have been employed to tackle and enhance the performance on many tasks such as skin cancer detection instead of training models from scratch. However, the existing systems are unable to attain substantial levels of accuracy. Therefore, we propose, in this paper, a robust skin cancer detection framework for to improve the accuracy by extracting and learning relevant image representations using a MobileNetV3 architecture. Thereafter, the extracted features are used as input to a modified Hunger Games Search (HGS) based on Particle Swarm Optimization (PSO) and Dynamic-Opposite Learning (DOLHGS). This modification is used as a novel feature selection to alloacte the most relevant feature to maximize the model’s performance. For evaluation of the efficiency of the developed DOLHGS, the ISIC-2016 dataset and the PH2 dataset were employed, including two and three categories, respectively. The proposed model has accuracy 88.19% on the ISIC-2016 dataset and 96.43% on PH2. Based on the experimental results, the proposed approach showed more accurate and efficient performance in skin cancer detection than other well-known and popular algorithms in terms of classification accuracy and optimized features.

## 1. Introduction

Among the common spreading cancers worldwide, skin cancer can threaten human lives and causes serious danger. Skin cancer can affect the cells of the skin in any region of the body, especially sun-exposed areas. Based on the grown abnormal skin cell, skin cancer can be categorized into three types including the common type which is basal cell carcinoma, squamous cell carcinoma, and melanoma which is the less common type and the more dangerous compared to the other two types [1,2]. A significant challenge to facing skin cancer and preventing its negative impact on the skin is early detection which can be challenging, especially in its early stages. In addition, most people avoid periodic checks due to the lack of medical resources, far clinics, lack of specialists, or expensive diagnoses and treatments which can change the case of skin cancer to a more severe case and help the spread. The necessity of early detection, monitoring, and taking precautions seriously can decrease the dangerous complications rate and lower the physical effects [2,3].

Skin lesions can be examined by specialists or with the help of a diagnostic tool such as Dermoscopy which generates a dermoscopy image. Relying on specialists’ opinions only to examine the skin lesions can not be reliable in most cases where the need for dermoscopy images is highly important. However, dermoscopy images can suffer from various limitations, which make the interpretation difficult, including the existence of a highly trained expert, images can be complex, and the quality of the images can differ based on the device used to capture the image, affecting the appearance of the lesion. In addition, the captured area of the body in the dermoscopy image can affect the quality of the image in terms of the location, skin type, color, lighting, magnification, and skin thickness [4,5]. Thus, an automatic melanoma detection tool or algorithm based on dermoscopy images is valuable in improving skin lesions’ diagnosis and management rather than relying only on clinical expertise [3].

In computer vision, various studies have incorporated different features extracted from dermoscopy images to improve the detection accuracy of different skin cancer types including handcrafted features [3,6] and automatically learned features [7,8]. The commonly used algorithms to automatically learn and extract features uses Convolutional Neural Network (CNN) which can achieve remarkable performance on the detection task [7,8]. In addition, deep learning (DL) networks can require a large amount of data during the training phase. Thus, using transfer learning to fine-tune a pre-trained network on similar tasks can minimize the training complexity, fast convergence, and lower the training time [9]. Besides, pre-trained DL models can be employed as feature extractors without further training if they are already trained on similar tasks or related domains [10]. The learned features by the DL model can hold some noise which affects the final classification accuracy due to the presence of nonrelevant features of the high dimensionality of the learned features. Thus, optimization techniques such as metaheuristic algorithms can offer a great solution in the case of performing feature selection and only selecting the most relevant features to boost detection accuracy [11,12].

The proposed framework incorporates deep learning and optimization algorithms. At first, a deep learning model is proposed to process the inputted skin cancer images and learn to extract relevant and meaningful representations automatically without human intervention. At this stage, a pre-trained version of MobileNetV3 is used and fine-tuned to extract the image embeddings. Thus, rather than relying on raw images, we extract new input image representations that serve as the input of the feature selection algorithm. Second, a novel feature selection algorithm is proposed to filter each image embeddings and select only the most relevant attributes to improve the overall framework skin lesion recognition performance and reduce the representation dimensionality to fit on edge devices. This FS method depends on improving the behavior of a new metaheuristic technique named Hunger Games Search (HGS), called Dynamic-Opposite Learning (DOL). The aim of the developed FS, named DOLHGS, is to find the relevant features from the extracted ones using MobileNetV3. This is achieved through integrating the operators of HGS and DOL. The metaheuristic approaches have generally established their performance as FS techniques among the traditional wrapper or filter FS methods. These traditional FS methods such as exhaustive search, random search, and greedy search suffer from premature convergence, and high computational. So, the MH are used since they are efficient and effective techniques. To validate the developed framework, two real-world datasets which are PH2 and ISIC-2016 were used to evaluate and analyze the performance and report extensive experimental results.

The following summarizes the significant contributions of this study:

1. A pre-trained deep learning is used to learn and extract new representations for skin cancer images.
2. A novel FS algorithm is proposed to reduce the dimensionality of extracted features and improve the overall performance by determining the relevant features.
3. Two real-world datasets are used to validate and compare the proposed method to well-known methods.
4. A more general framework is suggested to integrate the proposed method into the system.

The remainder of the paper is laid out as follows: Section 2 includes a review of prior relevant works. Section 3 presents the background of the convolutional neural network as feature extraction and the Hunger Games Search (HGS). Section 4 contains the details of the suggested framework. The outcomes of the experiments are analyzed and discussed in Section 5. Finally, Section 6 gives our concluding remarks and suggests possible future developments.

## 2. Related Works

Medical classification is a crucial area of study due to its ability to assist in medical diagnosis. Recently, researchers have used deep learning and FS optimization to enhance classification performance on the Internet of Medical Things. This section discusses the classification of medical images using deep learning and FS optimization algorithms.

### 2.1. Deep Learning-Based Medical Images

Deep Learning techniques have recently demonstrated excellent performance in natural language processing [13,14,15] and image processing [16,17]. Convolutional Neural Networks (CNN) has become a popular deep learning model, where the use of CNNs in object recognition has recently shown encouraging results, and they have become a key study field in medical image analysis categorization [11,18]. Moreover, machine learning algorithms are applied in the majority of skin cancer detection investigations, however deep learning algorithms are used in a small number of skin lesions categorization investigations [19]. Otherwise, deep learning techniques need big quantities of well-labeled training examples [12,20].

As a result, to address the problem, transfer learning has been implemented. Due to its ability to successfully address the flaws of reinforcement learning and supervised learning, transfer learning is becoming increasingly popular [21]. According to the traditional view, in the test, machine learning is pre-trained and then refined for use with specific data. Transfer learning can be used to fine-tune already pre-trained models on new related tasks which shows to be efficient in improving many DL tasks rather than training the DL model from scratch [22]. It is theoretically possible to create effective objectives relevant way only a limited subset of training samples and by transferring information learned from other domains and activities. A deep learning algorithm on input from another set of medical centers (perhaps from different regions) may also result in a demographic incompatibility between the training and testing due to differences in patient features, as well as differences in imaging methods. However, this could result in very weak results [23]. Learning the algorithm with relevant data from the health center where the algorithm is planned to be utilized (target data) is, therefore, an essential undertaking that faces the issue of dealing with extremely small labeled datasets [23]. Several annotated source domains with considerable inconsistencies profit from a unique feature-based technique [24]. It’s also capable of multi-class categorization and routinely produces positive results.

To classify skin cancer images, researchers recently included a pretrained CNN and transfer learning rather than building a CNN from scratch with random initialization parameters [25]. This pre-training significantly decreased CNN’s training time, which led to an accuracy of 84.8% across five categories. In this situation, transfer learning enables models to be used on various and related tasks since they are learned on a single task or huge dataset. In [26], they used a deep learning-based method to find melanoma early. They applied a proposed VGGNet structure and a transfer learning approach to the skin lesion classification algorithm. The suggested method’s sensitivity on the ISIC Archive dataset was 78.66%. In [27], the performance of the classification algorithm for identifying skin lesions was assessed using an augmented and non-augmented dataset.

Small data sets are one problem that prevents the successful detection of skin cancer. Therefore, for the purpose of effective detection of melanoma skin cancer, Abayomi et al. [19] proposed a new method to augment the data. In order to facilitate the automatic identification of melanoma, Kadry et al. [16] suggested a scheme that applied a CNN-based method. To extract the skin melanoma from the dermoscopy image, they used the VGG-SegNet model. After that, the segmented skin melanoma and the ground truth are compared, and the key performance indices are calculated. In Ref. [17], they presented the DenseNet-based UNET model for efficient melanoma segmentation. To determine representative features, they added the DenseNet model to the UNET encoder unit. However, they noted that deep learning techniques might be advantageous without sufficient data. Recent years have seen a significant increase in the use of CNNs in medical image processing because of their strong feature representation capabilities. Tang et al. [28] proposed a multi-stage model based on an extremely deep residual network for fully automated skin cancer identification in medical images. In contrast to earlier approaches, they developed a classification method in the lightweight melanoma prediction model to improve feature selection, reduce computational, and limit the number of hyperparameters.

Lately, it has become clear that the Internet of Medical Things (IoMT) innovation is perfect for building intelligent systems that can diagnose illnesses precisely, just like experts do [29]. In Ref. [6], vital medical systems have benefited from the development of IoMT technology. It is now available to doctors in a variety of settings, improving their ability to diagnose patients without affecting subjective factors. In contrast, the problem of unbalanced data between uncommon and common illnesses was still there for any approach. This problem consequently led to bad performance. However, in the medical field, the classifier should be confident in its precision with a high percentage when identifying the cancer type. A previous study found that timely melanoma recognition is essential for giving patients the right care. We are therefore working to enhance melanoma medical diagnostics.

### 2.2. Medical Images Classification Using FS Optimizers

FS optimizers have been used to successfully resolve a wide range of challenging optimization problems in the real world. They can effectively move through the solution space because they can use a list of candidate solutions instead of just one. Meta-heuristic optimization techniques, therefore, outperform other optimization techniques. Task scheduling in the IoMT has benefited from the development of numerous meta-heuristic techniques [30], such as particle swarm optimization (PSO) [31], Multi-Verse Optimizer (MVO) [32], and BAT Algorithm [33]. A few experience local minima and convergence rates, notably when a sizable solution space is involved [34]. This restriction frequently leads to ineffective task scheduling techniques, negatively affecting the system’s performance. Consequently, there is an important need for an optimal global solution to the scheduling algorithm problem. So, this study aims to identify the best solutions that enhance the rate of convergence, as demonstrated in the following sections.

## 3. Background

This section provides the basic information on Efficient neural networks, Hunger Games Search, and Dynamic-Opposite Learning.

### 3.1. Efficient Neural Networks

Recently, researchers have proposed various convolutional neural network designs and architectures to enhance efficiency based on time and space. Depthwise convolutions such as NASNet [35], MobileNets [36,37], MnasNet [38], ShuffleNets [39], and EfficientNet [40] can fullfil the aforementioned requirements. Thus, the networks are increasingly used in different applications [41,42,43]. The MobileNetV3 [37] is a computationally efficient and optimized architecture for the classification of images and the deployment on embedded systems or IoT devices. In addition, the traditional convolutional layers is replaced with depthwise and pointwise convolutional layers in the MobileNetV3 offers the power of learning more complex representations, thus, achieving remarkable performance on the image classification task. In this section, we will briefly introduce the recently proposed MobileNetV3 as we will use it in our framework.

Recently, Howard et al. [37] introduced MobileNetV3 which came to enhance the previous versions of MobileNet (V1 and V2) with the ability to learn the optimal kernel size using network architecture search (NAS) technique (NetAdapt algorithm). The MobileNetV3 is implemented using the following components: the depthwise separable convolutional layer and the global average pooling layer. The depthwise convolutional layer comprises the depthwise convolutional kernel, the batch normalization layer and the ReLU activation function. In addition, the MobileNetV3 combines different modules from previous versions of MobileNet in the Inverted Residual Block (MBConv Block). These modules can include the Squeeze-And-Excite block [38] and a modified nonlinearity called hard swish introduced in [44,45].

**Figure 1:**
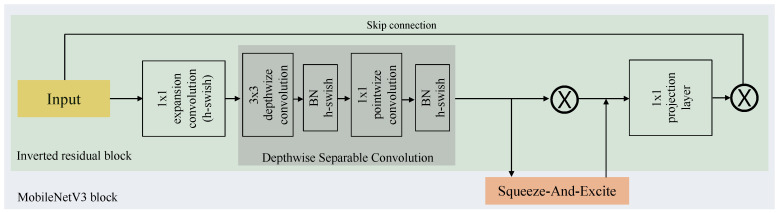
The structure of MobileNetV3 block.

In MobileNetV3, the depthwise separable convolutional layer can be seen as the core building block of the network as shown in Figure 1. The aim of using the depthwise separable convolutional is to replace the traditional convolution layer with a factorized version, reducing the model size. The block of the depthwise separable convolutional layer is composed of two layers which are: (1) depthwise convolution which applies a single convolutional filter to each input channel, (2) and a 1×1 convolution (pointwise convolution) to compute the linear combinations of the input channels and generate new feature maps.

Meanwhile, inspired by the intuition behind the bottleneck blocks where several blocks can contain an input followed by a sequence of bottlenecks [46]. The MobileNet architecture implements an inverted residual connection design in the bottleneck block to improve the model performance and lower memory usage. The inverted residual block shown in Figure 1 has been implemented in MobileNetV2 with a residual structure to build a robust feature descriptor and learn more complex nonlinear relationships with feature expansion. The inverted residual block is built using: (1) 1×1 expansion convolution to enhance the model performance with fewer calculations, (2) depthwise separable convolution, (3) and a 1×1 projection layer with residual connection.

### 3.2. Hunger Games Search

Yutao and Huiling [47] proposed the Hunger Games Search (HGS) optimizer which simulates the behaviors of animals and hunger. The mathematical modeling of HGS starts with a set of *N* solutions (*X*), then get the values of the fitness value ($Fit_{i}$) of *X*. Then the phase of modernization is performed by the following equations:

(1) $$X=\left\{\begin{array}{c} X\left(t\right)\times \left(1+rand\right),\;r_{1}<l\;\\ W_{1}\times X_{b}-R\times W_{2}\times \left|X_{b}-X\left(t\right)\right|,\;r_{1}>l,\;r_{2}<E\;\\ W_{1}\times X_{b}+R\times W_{2}\times \left|X_{b}-X\left(t\right)\right|,\;r_{1}>l,\;r_{2}>E \end{array}\right.$$

where $r_{1}$, $r_{2}$ and rand are random numbers. *R* is a random value from [−a,a] and it is defined as:

(2) $$R=2\times rand\times s-s,\;\;\;s=2\times \left(1-\frac{t}{T}\right)$$

The *E* is defined as:

(3) $$E=\mathrm{sech}\left(\left|Fit_{i}-Fit_{b}\right|\right)$$

Also, $Fit_{b}$ stands for the finest value. Whereas $W_{1}$ and $W_{2}$ are the hunger weights defined as:

(4) $$W_{1}=\left\{\begin{array}{c} H_{i}\times \frac{N}{SH}\times r_{4},\;r_{3}<l\;\;\\ 1,\;\;\;\;\;r_{3}>l \end{array}\right.$$

(5) $$W_{2}=2\left(1-e^{\left(-|H_{i}-SH|\right)}\right)\times r_{5}$$

where $r_{3},\;r_{4}$ and $r_{5}$ are random numbers, and SH stands for the hungers feeling summation that defined as:

(6) $$SH=\sum_{i}H_{i}$$

As well as, the variable $H_{i}$ is defined as:

(7) $$H_{i}=\left\{\begin{array}{c} 0,\;\;\;\;\;\;\;Fit_{i}=Fit_{b}\;\\ H_{i}+H_{n},\;\;\;otherwise \end{array}\right.$$

where $Fit_{b}$ is the finest value and $Fit_{i}$ is the fitness of $X_{i}$. The new hunger $H_{n}$ is formulated as:

(8) $$H_{n}=\left\{\begin{array}{c} LH\times \left(1+r\right),\;\;\;\;\;\;TH<LH\;\\ TH,\;\;\;otherwise \end{array}\right.$$

(9) $$TH=2\frac{Fit_{i}-Fit_{b}}{Fit_{w}-Fit_{b}}\times r_{6}\times \left(UB-LB\right)$$

In addition, the objective function has a worse value given by $Fit_{w}$, also $r_{6}\in \left[0,1\right]$ is a random value which can tell that the hunger has positive or negative actions according to several factors (Algorithm 1).
**Algorithm 1** Steps of HGS1:Initial the number of iterations *T*, size of population *N*.2:Build the population *X*.3:**while** t≤T **do**4:    Compute the fitness value for $X_{i}$.5:    Allocate the value of $X_{b},$ $Fit_{W}$, and $Fit_{b}$.6:    Enhance $H_{i}$ using Equation (7)7:    Update $W_{1}$ and $W_{2}$ based on Equation (4) and Equation (5), respectively.8:    **for** doi=1:N9:         Enhance *R* using Equation (2)10:        Enhance *E* using Equation (3)11:        Enhance $X_{i}$ using Equation (1)12:    t=t+113:Return $X_{b}$.

### 3.3. Particle Swarm Optimization

Kennedy and Eberhart [48] created Particle Swarm Optimization (PSO), which replicates the evolution of the understanding of a social activity [49]. First, random particles are created and their positions ($x_{i}$) and speeds ($v_{i}$) in a given dimension *j*-th are determined. Particle locations [49,50] are updated using Equations (10) and (11).

(10) $$x_{ij}^{\left(t+1\right)}=x_{ij}^{\left(t\right)}+v_{ij}^{\left(t+1\right)}$$

(11) $$v_{ij}^{\left(t+1\right)}=wv_{ij}^{\left(t\right)}+c_{1}r_{1}\left(x_{ij}^{p(t)}-x_{ij}^{\left(t\right)}\right)+c_{2}r_{2}\left(x_{j}^{g(t)}-x_{ij}^{\left(t\right)}\right)$$

where $x_{ij}$ refers to the location of particle *i* in dimension *j*, $v_{ij}$ is the *i*-th velocities in the *j*-th dimension, *t* is the current state, and *w* is a weight vector used to accelerate population merging. The acceleration coefficients $c_{1}$ and $c_{2}$ are constants. $x_{ij}^{p(t)}$ denotes the particle *i*’s best prior position at dimension *j*, while $x_{j}^{g(t)}$ indicates the optimum global location in dimension *j*. The random values on $r_{1}$ and $r_{2}$ are ∈[0,1].

This process is repeated until the stopping requirements (for example, a set number of iterations) are met. The PSO are described in Algorithm 2.
**Algorithm 2** Algorithm of PSO1:Input *N* the number of solutions and total number of generations.2:Set the population to its initial state.3:**repeat**4:    **for** i=1 to *N* **do**5:         Compute the fitness value for *X*.6:         **if** Fitness value of updated $X_{i}$< its PBest **then**7:           Using updated $X_{i}$ as current solution.8:         Find the best solution with the best fitness (GBest) overall *X*.9:         Using Equation (11) to update the velocity.10:       Equation (10) used to update $X_{i}$11:**until** The termination requirement has been reached.

### 3.4. Dynamic-Opposite Learning

This section gives the basic steps of the Dynamic-Opposite Learning (DOL) optimization strategy. First, we will discuss the traditional Opposition-based learning (OBL) strategy [51] that is used to improve different optimization approaches [52,53]. OBL strategy is applied to construct a new opposition solution to the current one. This strategy seeks to allocate the best solution that improves the convergence rate.

For the real number X∈[U,L], its opposite value $X^{o}$ is computed as:

(12) $$X^{o}=U+L-X$$

Opposite point [52]: Consider *X* = [$X_{1}$, $X_{2}$, …, $X_{Dim}$] be a solution in a space of Dim-dimensional, and $X_{j}$ [$U_{j}$,$L_{j}$]. So, the opposite point $X^{o}$ of *X* is given as:

(13) $$X_{j}^{o}=UB_{j}+L_{j}-X_{j},\;\;where\;j=1,\ldots ,Dim.$$

Besides, the best of the two points $X^{o}$ and *X* is selected based on the fitness value and the other one is ignored.

Similar to $X^{o}$, the Dynamic opposite value $X^{DO}$ of the *X* is defined as:

(14) $$X^{Do}=w\times r_{8}\left(r_{9}\times X^{o}-X\right)+X,w>0$$

where *w* is the weighting factor. The $r_{8}$ and $r_{9}$ refer to random numbers.

So, the Dynamic opposite point $X_{j}^{DO}$ of point *X* = [$X_{1}$, $X_{2}$, …, $X_{Dim}$] is defined as:

(15) $$X_{j}^{Do}=X_{j}+w\times rand\left(rand\times X_{j}^{o}-X_{j}\right),w>0$$

Therefore, DOL optimization starts by generating the initial solutions $X=(X_{1},\ldots ,X_{Dim}$ and compute its dynamic opposite solution $X^{Do}$ using Equation (15). Then according to the fitness value, the best of them (i.e., $X^{Do}$ and *X*) is determined and the other one is removed.

## 4. Proposed Model

The structure of the developed model is discussed in this section. First of all, the skin cancer images are collected. In case the aim is to train the developed model, there are three steps; the first step is to apply the DL model to extract the feature. Followed by the second step that aims to allocate the relevant features based on the modified HGS, named DOLHGS since it depends on DOL. The third step is to train the classifier. However, if the aim is to predict the case of the collected image, then the trained model is used directly. The details of the developed model are given in the following sections.

### 4.1. Deep Learning for Feature Extraction

This section describes the discriminative learning model used in the experiments to perform feature extraction. Convolutional neural networks and their variants have flexible architectures known for their success in applications such as image recognition [9,54]. Compared to previous studies, we introduced the usage of swarm optimization on top of features extracted from a pre-trained DL model to improve recognition accuracy, extracting the most relevant features, and reducing the number of features.

In our experiments, a pre-trained MobileNetV3 [37] on the ImageNet dataset is used as the backbone for the feature extraction phase in our framework. Based on resource capacity, there are two variants of MobileNetV3 which are MobileNetV3-Large and MobileNetV3-Small. This study used MobileNetV3-Large and adapted it to skin cancer detection tasks via fine-tuning the pre-trained model on different skin cancer datasets (ISIC-2016 and PH2). MobileNetV3 combines several building blocks, including depth-wise separable convolutions, linear bottleneck, and inverted residual structure. The depth-wise separable convolutions have been introduced in MobileNetV1 [36] to replace the traditional convolution layers, lower the model’s size, and make it easier to execute on mobile devices.

Transfer learning is used as the main mechanism in the feature extraction phase with the following steps:

(1) Replacing the two last output layers in MobileNetV3 with dense connected blocks including two 1×1 convolutions for feature extraction and classification, respectively;
(2) Fine-tuning the modified MobileNetV3 on the skin cancer dataset;
(3) Extracting the corresponding feature vector of each image from the convolution layer added to the MobileNetV3 model; where the extracted features for each image are flattened into a vector of size 128.
(4) Later, the extracted features for each image are fed to the feature selection part in our framework.

The 1×1 convolution can be seen as a multilayer perceptron (MLP) that can perform operations such as dimensionality reduction and applying non-linearity after convolutions. The added 1×1 convolution block receives input channels of size 960 from the last layer feature extractor in MobileNetV3 and outputs 128 channels (used for feature extraction) with a corresponding kernel of size 1. The last 1×1 convolution block acts as a fully-connected layer for image classification.

Table 1 shows the architecture of MobileNetV3 used as a feature extractor backbone for skin cancer detection. The image embedding (features) is collected for each image in the dataset and fed to the feature selection phase. The adapted MobileNetV3 was fine-tuned for 50 epochs before performing feature extraction with a batch of size 16. RMSprop, a form of stochastic gradient descent, trains the network with the learning rate set to 0.0001. The ensure model generalization and overcome the overfitting, data augmentation is performed on the training set using several image transformations including: resizing images into 224×224, random crop, color jitter, random horizontal flip, and random vertical flip.

### 4.2. Steps of DOLHGS Feature Selection Algorithm

**Figure 2:**
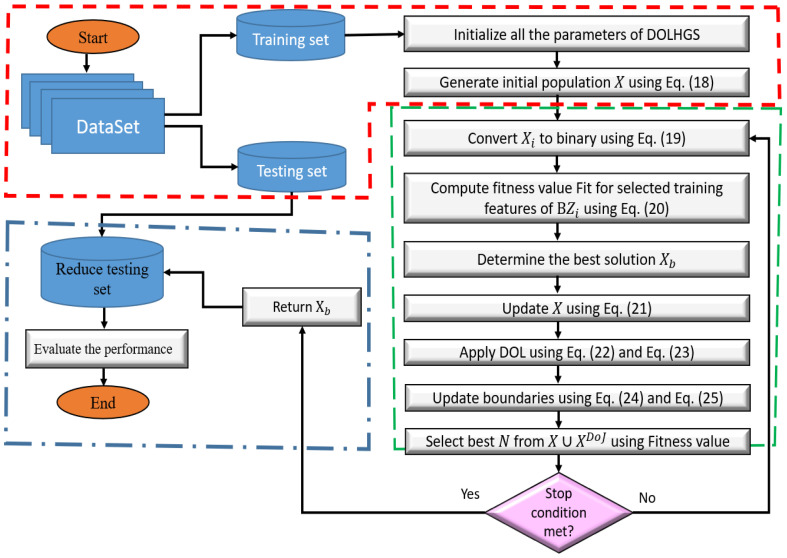
Diagram showing the suggested melanoma detection methodology.

Within this section, we introduces the steps of the improved HGS, as the FS method, based on DOL. The general steps of the proposed FS approach, named DOLHGS, are given in Figure 2. The first step in the developed DOLHGS is to generate the set of *N* agents *X* which represents the solutions to the FS problem. This process is performed using the following equation:

(16) $$X_{i}=rand\times \left(U-L\right)+L,\;i=1,2,\ldots ,N,\;j=1,2,\ldots ,Dim$$

In Equation (16), *U* and *L* are the boundaries of search domain. Dim refers to the dimension of the given data (i.e., the number of features). Then we obtain the Boolean version of $X_{i}$ and this is achieved using Equation (17).

(17) $$BX_{ij}=\left\{\begin{array}{cc} 1 & if\;X_{ij}>0.5\\ 0 & otherwise \end{array}\right.$$

The next process is to compute the fitness value of $X_{i}$ as defined in Equation (18).

(18) $$Fit_{i}=\lambda \times \gamma_{i}+\left(1-\lambda \right)\times \left(\frac{|BX_{i}|}{Dim}\right),$$

In Equation (18), $\left(\frac{|BX_{i}|}{Dim}\right)$ is the ratio of selected features. $\gamma_{i}$ is the classification error (we used KNN at K=5). λ stands for a parameter that applied to balance between $\left(\frac{|BX_{i}|}{Dim}\right)$ and $\gamma_{i}$.

Since the initial population has the largest effect on the convergence of the agents towards the optimal solution, the DOL is applied to the initial population *X* using Equation (15). Then computing the fitness value for each $X_{DO}$, select the best *N* agents from $X\cup X_{DO}$ according to the fitness value. Thereafter, the best solution $X_{b}$ is determined which has the smallest fitness value $Fit_{b}$.

Thereafter, the value of $X_{i}$ is updated through using either the operators of HGS or PSO. This is achieved according to the probability $Pr_{i}$ of each $X_{i}$. In the case of $Pr_{i}>0.5$, the operators of HGS are used as defined in Equations (1)–(7); otherwise, operators of PSO are used as in Equations (10) and (11). This updating process is formulated as:

(19) $$X_{ij}=\left\{\begin{array}{cc} Equations\;(1)–(7) & if\;Pr_{i}>0.5\;\\ Equations\;(10)\;\mathrm{and}\;(11) & otherwise \end{array}\right.$$

where $Pr_{i}\in \left[0,1\right]$ is the random probability value used to make the operators of HGS and PSO competitive during updating the solutions.

Thereafter, the DOL is applied to the current updated population *X*. However, since the DOL can take more time, it will be applied if the probability $Pr_{DO}$ is smaller than 0.5 as formulated in the following formula:

(20) $$X_{ij}=\left\{\begin{array}{cc} X_{ij} & if\;Pr_{DO}>0.5\;\\ X_{ij}^{DoJ} & otherwise \end{array}\right.$$

(21) $$X_{ij}^{DoJ}=w\times rand\left(rand\times X_{ij}^{o}-X_{ij}\right)+X_{ij},w>0$$

In Equation (21), $X_{ij}^{o}$ is given in Equation (15). In addition, the search space [U,L] is dynamically updated during the searching process as:

(22) $$L_{j}=min\left(X_{ij}\right)$$

(23) $$U_{j}=max\left(X_{ij}\right)$$

The best solutions from $X\cup X^{DoJ}$ are selected based on the fitness value.

The next process is to check the terminal criteria and when they are met then the algorithm return $X_{b}$. Otherwise, the updating stage are conducted again.

### 4.3. Framework of the Developed Skin Cancer Detection

The general structure of the developed platform illustrated in this section, In general, the proposed platform comprises two systems (i.e., training and testing). The training system can be used to fine-tune the developed framework from Section 4.1 and Section 4.2. In this case, we use the pre-trained feature extraction model and benefit from the lightweight and fast model to make the process faster. In this study, the mobilenetv3 architecture used for feature extraction is well-known for its compatibility and low resource usage on embedded systems. After the feature extraction phase, the proposed DOLHGS as a light and robust feature selection technique is used to minimize the features representation space and only keep the most relevant features from each processed image. Reducing the dimension of the feature will help the classification system, which is a basic k-nearest neighbors (KNN) model, perform faster training and provide a classification decision in a reasonable time window. The second system in the proposed platform uses the best pre-trained version of the proposed training system to make predictions on the fly without the need to train the system again. As a result, the system will be provided the final decision alongside different evaluation measures such as accuracy, F1-score, and more.

## 5. Experiments and Results

This section discusses the experiments performed and their results in order to propose a highly effective and efficient approach for melanoma detection.

### 5.1. Description of Datasets

**Figure 3:**
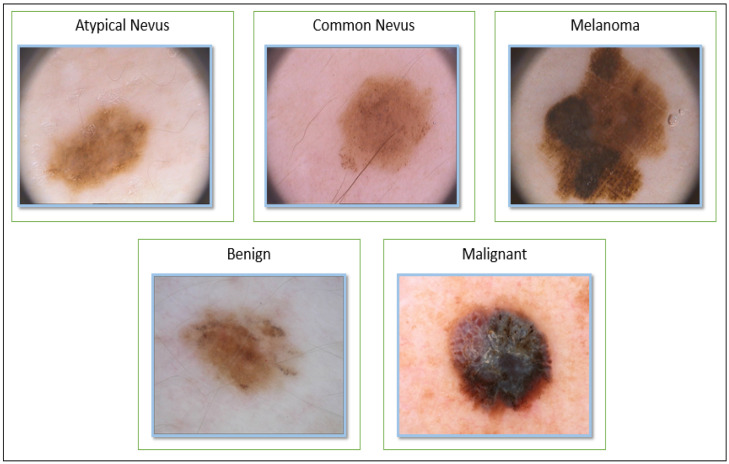
Example images for melanoma detection task from two datasets. The bottom row shows the ISIC-2016 challenge dataset and the top row displays the PH2 dataset.

For our experimental evaluation, two datasets of dermatoscopic images are used to conduct skin cancer classification tasks: the International Skin Imaging Association 2016 challenge dataset (ISIC-2016) [55] and Hospital Pedro Hispano dataset (PH2) [56]. The ISIC-2016 dataset contains 1179 images, divided into two categories: Approximately 80% of the dataset is benign and the rest is malignant. This database is public for download at the website https://challenge.isic-archive.com/data (accessed on 13 March 2023). Detailed information about the ISIC-2016 dataset can be found in [55]. The PH2 database was divided into 3 classes consisting of 200 images, as presented in [56]. This database used is available for free download at http://www.fc.up.pt/addi/ph2%20database.html (accessed on 13 March 2023). The dermoscopic images are 8-bit RGB, compressed in JPEG format with 768 × 560-sized color images. Furthermore, some samples of the images for the two datasets are represented in Figure 3. Table 2 describes more information about the datasets.

### 5.2. Performance Measures

Our proposed classification method is assessed by measuring Precision (P), Recall (R), Accuracy (AC), F1-measure (F1) and Performance Improvement Rate (PIR).

(24) $$R=\frac{TP}{TP+FN}$$

(25) $$P=\frac{TP}{TP+FP}$$

(26) $$F1=\frac{2*P*R}{P+R}$$

(27) $$AC=\frac{TP+TN}{TP+TN+FP+FN}$$

(28) $$PIR\left(\%\right)=\frac{\left(AC-AC^{\prime }\right)}{AC^{\prime }}*100$$

In Equations (24)–(27), false Positives (FP) are non-melanoma photos that have been mistakenly classified as melanomas. True Negative (TN) refers to the proportion of accurately identified non-melanoma images. False Negative (FN) images are those in which non-melanoma images are mistakenly classified as melanomas. Recall is a metric used to determine how many labels the system finds. Precision is a way to gauge how many labels the system assigned correctly. Precision and recall are necessary for the F1-measure to produce accurate results. Accuracy determines the identification rate of the system. Finally, the performance improvement rate is an indicator that evaluates, in relation to other literature scheduling methods as defined in Equation (28) the percentage of improved performance on each technique proposed. AC′, and AC are the accuracy ratings derived from the associated by the suggested algorithm and the comparison approach, respectively.

### 5.3. Results and Discussion

In this study, the two datasets are randomly split into training and testing sets. A comparative study of the proposed work with the existing work for the classification of melanoma on the PH2 dataset and the ISIC-2016 dataset are conducted as described in the following sections.

#### 5.3.1. Comparison with FS Methods

This section confirms the performance of the newly proposed DOLHGS algorithm, which has been experimentally tested on the ISIC-2016 and the PH2 challenge dataset. The developed algorithm is compared to the Multi-Verse Optimizer (MVO) [57], Whale Optimization Algorithm (WOA) [58], Particle Swarm Optimization (PSO) [48], Bat Algorithm (BAT) [59], and Firefly Algorithm (FFA) [60].

Different metrics evaluate these optimization algorithms to address challenging numerical optimization problems. Table 3 illustrates the parameters of each method. The number of search agents is 50 and the number of iterations is 1000.

After training with the ISIC-2016 dataset and the PH2 dataset, the outcomes of feature selection algorithms are summarised in Table 4. For both datasets, the table shows the results of DOLHGS with MVO, PSO, WOA, FFA, BAT, and HGS optimization algorithms using four separate measurements.

For the ISIC-2016 dataset, the results demonstrate the ability of DOLHGS to achieve the best value for all metrics. WOA and BAT are also the second and third most effective, respectively, with the best accuracy, recall, and precision, respectively. On all four measures, MVO had the worst performance. In the second dataset, DOLHGS has the highest level of stability, followed by WOA, PSO, and HGS. On the other hand, MVO is the least efficient. Finally, as can be seen from this table, DOLHGS gives the best performance and outperforms the other algorithms since it received the best results in terms of optimization performances for the two datasets. In this table, the best performances are bolded.

**Figure 4:**
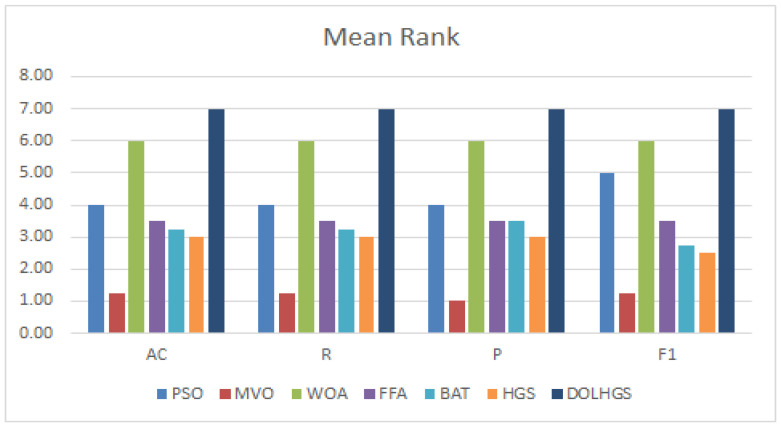
The mean rank for each algorithm.

Moreover, the Friedman (FD) test as a nonparametric is used to analyze the difference between the DOLHGS and others, in which the mean rank value is computed. In [61], the FD test is applied to check whether there is a significant difference between other methods overall the datasets. The results of FD are given in Figure 4 for each approach. Based on to the results of FD, the DOLHGS has a better mean rank value than the other approaches according to ISIC and PH2 datasets. In addition, the p-value is smaller than 0.05 for the methods.

In the ISIC dataset, we noticed that the DOLHGS and WOA have a mean rank of 7 and 6, respectively. BAT and PSO have almost the same mean level. However, FFA delivers more significant results than HGS, averaging 3.25, with HGS 2. Lastly, MVO is less than others, with a mean rank of 1. From the FD test results for the PH2 dataset, we also noticed that DOLHGS is better than others in its mean rank of 7, then WOA, with a mean level of 6. The other algorithms, FFA and HGS, equal the mean rank (i.e., 3.75). Finally, the lowest mean ranking is BAT and MVO.

**Figure 5:**
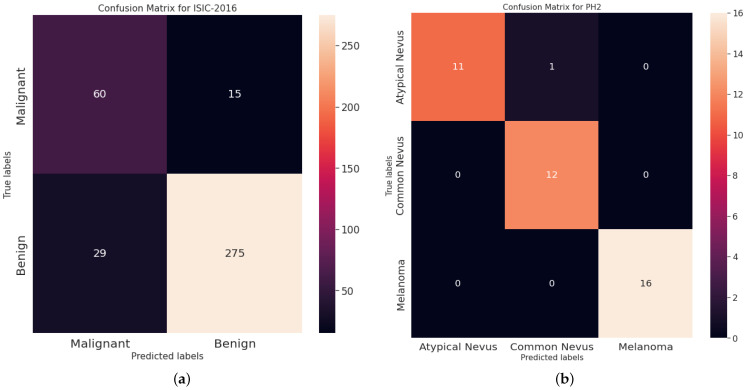
Confusion matrices obtained of DOLHGS for skin cancer classification: (**a**) ISIC-2016 and (**b**) PH2.

**Figure 6:**
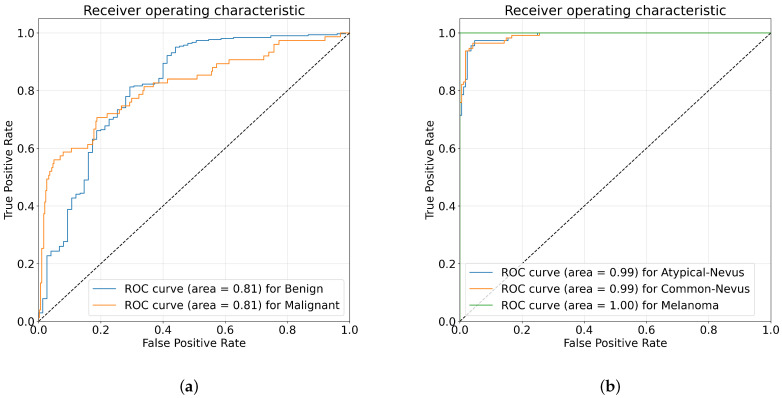
ROC curves obtained of the DOLHGS for skin cancer classification: (**a**) ISIC-2016 and (**b**) PH2.

For more analysis, Figure 5 and Figure 6 illustrates the ROC curves and the confusion matrices obtained using the proposed DOLHGS approach for skin cancer classification. It can be noticed the efficiency of the developed DOLHGS for the two datasets.

#### 5.3.2. Comparison with Previous Works

A lot of effort is being put into developing high-accuracy technologies for melanoma diagnosis. We compared our method against other existing state-of-the-art algorithms that have been verified on the same datasets to provide a fair comparison. Table 5 and Table 6 compare the performance of various approaches for melanoma detection on the PH2 and ISIC datasets, respectively. For the ISIC dataset, we conducted a thorough comparison with advanced melanoma detection technologies, which include: Based on separation first and then recognition [62], based on feature fusion [63], Fisher coding and deep residual network are coupled [9], multi-CNN collaborative training model [10], using ensemble method [64], combining Fisher Vector and multi-CNN fusion [54]. A fine-grained categorization principle is used to discriminate features [4]. For the other dataset (i.e., PH2), different methods have been developed. For example, the artificial neural networks [65] is used to construct a decision-support system. Kernel sparse model-based strategy to represent features in a high-dimensional feature space was introduced by [66]. In [67], U-Net was offered as a tool for automatically classifying melanomas. In [6], transfer learning and CNN were used in their framework. For learning features, a hierarchical framework based on two-dimensional superpixels and ResNet-50 was presented in [68].

From another point of view, The PIR(%) based on the accuracy of the proposed DOLHGS approach as it relates to other existing state-of-the-art algorithms is presented in Table 5 and Table 6 on the ISIC and PH2 dataset, respectively. For the execution of the ISIC dataset, DOLHGS shows 3.05%, 3.62%, 1.56%, 2.14%, 0.22%, 1.56%, and 0.67% accuracy improvements over the CUMED, BL-CNN, DCNN-FV, MC-CNN, KNORA-E, MFA, and FUSION methods, respectively. Furthermore, the DOLHGS algorithm outperforms the ANN, Kernel Sparse, Dense-Net201 + SVM, DenseNet201 + KNN, and NB techniques for the PH2 dataset (shown in Table 6) by 4.08%, 3.04%, 4.59%, 3.39%, and 1.07%, respectively. That is, DOLHGS outperforms the other approaches significantly.

In summary, our method is capable of removing extraneous features from high-dimensional images generated by CNN models. However, the major limitation of this framework, both the time and the memory are high levels of complexity. Our future work will focus on lowering complexity and enhancing the efficiency of the suggested framework, among other things. In order to increase our method’s performance, more augmentation approaches can be investigated in the future study. Finally, emphasize the potential role of IoMT in reducing healthcare costs associated with skin cancer management. By enabling early detection and personalized treatment, IoMT can help to reduce the need for costly surgeries and other interventions, and can help to optimize the use of healthcare resources.

## 6. Conclusions

This paper has presented an alternative skin cancer detection method. The developed method depends on MobileNetV3 architecture for feature extraction tasks using fine-tuning techniques on skin cancer datasets to learn more complex representations. In addition, allocate the relevant features from the extracted image representation according to a novel FS technique based on metaheuristic algorithms named DOLHGS. The improvement of the FS algorithm is performed using the operators of particle swarm optimization as a local search strategy and using dynamic opposite-based learning to improve the diversity of solutions. This leads to enhancing the convergence towards the optimal subset of relevant features. To evaluate the efficiency of the developed method a set of experiments have been performed using two real-world datasets named ISIC-2016 and PH2. The results show that the developed DOLHGS FS method is better than traditional FS methods. In addition, the comparison results with other state-of-the-art skin cancer detection methods illustrated that the developed IoMT approach is an effective method. Furthermore, the framework can be further optimized using other techniques to improve its performance and reduce its complexity such as employing neural architecture search and hyper-parameter optimization. Moreover, the developed technique can be applied to enhance the decision of diagnosis the skin cancer and this will help the expert to early detect the disease and treatments.

In future work, we plan to evaluate our approach on a larger number of datasets and to promote its use in clinical practice. Furthermore, combining numerous classifiers is an exciting field of study that can help researchers improve the effectiveness of their systems.

## Figures and Tables

**Table 1:** MobileNetV3 architecture as feature extraction backbone.

Input	Operator	Output	SE	NL	Stride
224×224×3	3×3 2d-Conv	16	FALSE	HS	2
112×112×16	3×3	16	FALSE	RE	1
112×112×16	3×3	24	FALSE	RE	2
56×56×24	3×3	24	FALSE	RE	1
56×56×24	5×5	40	TRUE	RE	2
28×28×40	5×5	40	TRUE	RE	1
28×28×40	3×3	40	TRUE	RE	1
28×28×40	3×3	80	FALSE	HS	2
14×14×80	3×3	80	FALSE	HS	1
14×14×80	3×3	80	FALSE	HS	1
14×14×80	3×3	80	FALSE	HS	1
14×14×80	3×3	112	TRUE	HS	1
14×14×112	3×3	112	TRUE	HS	1
14×14×112	5×5	160	TRUE	HS	2
7×7×160	5×5	160	TRUE	HS	1
7×7×160	5×5	160	TRUE	HS	1
7×7×160	1×1 2d-Conv	960	FALSE	HS	1
7×7×960	Adaptive average pooling	960	FALSE	-	1
1×1×960	Image embedding	128	FALSE	HS	1

3×3 2d-Conv: 3×3 depthwise separable convolution. NBN: no batch normalization. SE: Squeeze-And-Excite. NL: nonlinearity (HS denotes h-swish and RE denotes ReLU).

**Table 2:** Dataset Description.

Dataset	Skin Disease	# Training Images	# Testing Images	Total Images per Category
ISIC-2016	Malignant	173	75	248
	Benign	727	304	1031
	Total images	900	379	1279
$Ph^{2}$	Common Nevus	68	12	80
	Atypical Nevus	68	12	80
	Melanoma	34	6	40
	Total images	170	30	200

**Table 3:** Values of the parameters for each approach.

Algorithm	Value of the Parameters
DOLHGS	*EPSILON = 10 × 10${}^{-10}$, MIN-PROB = 0, MAX-PROB = −1*
WOA	*a = 2 to 0, a2 = −1 to −2*
BAT	*QMin = 0, QMax = 2*
MVO	*WEPMax = 1, WEPMin = 0.2*
PSO	*VMax = 6, WMax = 0.9, WMin = 0.2*
FFA	*Alpha = 0.5, BetaMin = 0.2, Gamma = 1*
HGS	*EPSILON = 10 × 10${}^{-10}$, POS = 0, F IT = 1*

**Table 4:** Average results for all experimental runs of each algorithm.

	ISIC				PH2			
	**AC**	**R**	**P**	**F1**	**AC**	**R**	**P**	**F1**
PSO	0.865699	0.865699	0.856919	0.852251	0.956429	0.956429	0.956949	0.956522
MVO	0.863325	0.863325	0.853915	0.849824	0.956071	0.956071	0.956575	0.956165
WOA	0.86781	0.86781	0.860512	0.853141	0.957143	0.957143	0.957592	0.957233
FFA	0.865435	0.865435	0.857003	0.85143	0.956429	0.956429	0.956918	0.956521
BAT	0.867018	0.867018	0.860102	0.851955	0.956071	0.956071	0.956581	0.956165
HGS	0.864908	0.864908	0.85652	0.850973	0.956429	0.956429	0.95694	0.956513
DOLHGS	**0.88185**	**0.87517**	**0.87633**	**0.87575**	**0.96429**	**0.97429**	**0.97699**	**0.97563**

**Table 5:** Accuracy and PIR (%) results of ISIC-2016 dataset.

Ref.	Year	Classification Model	AC (%)	PIR (%)
[62]	2016	CUMED	85.50	3.05
[63]	2017	BL-CNN	85.00	3.62
[9]	2018	DCNN-FV	86.81	1.56
[10]	2019	MC-CNN	86.30	2.14
[64]	2019	KNORA-E	88.00	0.22
[54]	2020	MFA	86.81	1.56
[4]	2020	FUSION	87.60	0.67
Our	present	DOLHGS	88.19	-

**Table 6:** Accuracy and PIR (%) results of the PH2 dataset.

Ref.	Year	Classification Model	AC (%)	PIR (%)
[65]	2017	ANN	92.50	4.08
[66]	2019	Kernel Sparse	93.50	3.04
[67]	2020	DenseNet201 + SVM	92.00	4.59
[6]	2020	DenseNet201 + KNN	93.16	3.39
[68]	2021	ResNet50 + NB	95.40	1.07
Our	present	DOLHGS	96.43	-

## Data Availability

The data are available from the authors upon request.
